# Circulating PAMM, a novel antioxidant and anti-inflammatory protein, is elevated in acute SCI

**DOI:** 10.1186/s12967-020-02304-0

**Published:** 2020-03-24

**Authors:** Leslie R. Morse, Nguyen Nguyen, Yan Xu, Prakash Jha, Ricardo A. Battaglino

**Affiliations:** Department of Rehabilitation Medicine, University of Minnesota School of Medicine, 500 Boynton Health Service Bridge, 410 Church St. SE, Minneapolis, MN 55455 USA

**Keywords:** PAMM, Biomarker, Inflammation, Reactive oxygen species, Spinal cord injury, Rehabilitation medicine

## Abstract

**Background:**

Peroxiredoxin activated in M-CSF stimulated monocytes (PAMM) is a novel protein produced by adipocytes with putative redox regulatory and anti-inflammatory properties. Because acute spinal cord injury (SCI) is associated with oxidative stress and neuroinflammation and because PAMM can be detected in systemic circulation, we hypothesized that acute neuro-trauma might induce changes in circulating PAMM expression. Specifically, we hypothesized that PAMM levels might vary based on the presence or absence of acute, traumatic SCI. We therefore investigated circulating PAMM levels in adults with and without acute traumatic SCI.

**Methods:**

We studied 105 men and women (54 with SCI and 51 without SCI). Participants with SCI were admitted for acute rehabilitation within 1 month after injury. Serum samples were obtained during hospitalization and stored at − 80 °C until batch analysis. Total PAMM was quantified by ELISA assay (MyBiosource, Cat. No: MBS9327247) with a detection limit of 0.25 ng/ml. Separate multivariate models including age, BMI, and injury severity were assessed to determine significant clinical predictors of change in PAMM levels.

**Results:**

When adjusting for BMI, age, and gender, mean change in PAMM levels were greatest in participants with motor complete SCI compared to able-bodied (1.65 ng/ml versus 0.94 ng/ml, p = 0.003). This model explained 26% of the variation in change in circulating PAMM levels.

**Conclusions:**

Our results suggest that PAMM may be a novel biomarker of neurological injury or of native anti-inflammatory responses to neurological injury. More work is needed to establish the role of PAMM and other adipocyte-derived factors in the acute response to neurotrauma.

## Background

According to the International Spinal Cord Society Prevention Committee, the global-incident rate of traumatic Spinal Cord Injury (SCI) is 23 cases per million [1]. SCI results in varying degrees of functional impairment based on the extent and location of trauma to the cord with high cost to both individual and society. The assessment of both injury severity and long-term neurological prognosis remain challenging after SCI [2]. While injury severity is routinely determined by neurological exam, there are instances where complete neurological testing cannot be performed (coma, intubated or nonverbal patient, presence of cast or other physical barrier). Furthermore, there is substantial variability in spontaneous neurologic recovery at 1-year post-injury and neurological examination does not differentiate between those who will experience recovery from those who will not. This variability in spontaneous recovery necessitates very large numbers for clinical trials that test novel neuroprotective therapies acutely after injury. There are no validated biomarkers of injury severity, neurological recovery, or response to neuroprotective therapy and this is considered a major limitation to both clinical care and to the development of adequately designed and powered clinical trials. Identification of a novel biomarker of spontaneous recovery would make clinical trial recruitment more economical and efficient. Identification of a biomarker that is easy to obtain and measure (i.e. circulates in blood) would represent a major advance in the field.

Trauma results in primary injury due to mechanical forces leading to compression or contusion of the cord. Subsequently, secondary injury occurs via multiple mechanisms including reactive oxygen/nitrogen species (ROS/RNS) generation, glutamate-mediated excitotoxicity, cellular necrosis and apoptosis [3–5], and the development of neuroinflammation. Oxidative stress occurs in primary injury due to cellular releases of cytoplasmic components and mitochondrial dysfunction and persists through the secondary injury phase due to severe neuroinflammation [5]. We recently identified a novel secreted protein with both antioxidant and redox-regulating activity which we called Peroxiredoxin-like 2 activated in M-CSF stimulated monocytes (PAMM) [6, 7], also known as adiporedoxin (Adrx) [8]. PAMM is broadly expressed across tissues, but is most highly expressed in white and brown adipose tissue. Furthermore, we demonstrated anti-oxidant and anti-inflammatory properties of PAMM in both in vitro and rodent in vivo models. Based on these properties, and because acute SCI is associated with neuroinflammation and can be detected in systemic circulation, we hypothesized that acute neuro-trauma might induce changes in circulating PAMM expression. We furthermore hypothesized that PAMM is a putative biomarker of acute SCI. We therefore investigated circulating PAMM levels in men and women with and without acute spinal cord injury.

## Materials and methods

### Subjects

We studied participants with traumatic SCI enrolled in our de-identified SCI biorepository. Participants were eligible for inclusion in the biorepository if they were 16 years or older, were inpatients receiving care for SCI at our rehabilitation hospital, and if they underwent a clinical blood draw during their inpatient stay. Serum samples were collected as discarded medical material at the time of each clinical draw obtained during the course of the rehabilitation stay, processed by the clinical laboratory, and banked for batch analysis. We collected samples from 141 individuals between December 10, 2012 and September 30, 2013. For the current analysis we selected for analysis a convenience sample of 54 adults with acute traumatic SCI. 51 age- and gender-matched men and women without SCI were studied as uninjured controls. The participants without SCI reported no medical conditions and were not taking medications at the time of study. Our Institutional Review Board approved this study.

### Variable definition

Clinical and demographic information including age, gender, motor level and completeness of SCI, medical history, date of clinical draw, height, and weight were obtained by medical record review at the time of enrollment and recorded in a de-identified fashion. Age and body mass index (BMI) were considered as continuous variables. SCI severity was considered in two categories: motor complete (AIS A/B) and motor incomplete (AIS C/D).

### Biochemical analyses

Plasma samples were drawn into an EDTA tube and immediately delivered to the core blood research laboratory at our facility. The samples were centrifuged for 15 min at 2600 rpm (1459×*g*) at 4 °C and stored at − 80 °C until batch analysis. Total PAMM was quantified by ELISA assay (MyBiosource, Cat. No: MBS9327247) with a detection limit of 0.25 ng/ml and an intra-and inter-assay CV of less than 15% as previously described [8].

### Statistical analysis

We used univariate and multivariable regression models to assess associations between clinical variables and circulating PAMM levels. Factors with a p-value of < 0.10 in the univariate models, as well as factors that were deemed clinically significant (age, gender, and/or BMI) were assessed in multivariable regression models. All analyses were performed using SAS 9.4 (SAS Institute, Inc., Cary, NC).

## Results

### Subject characteristics

Subject characteristics are presented in Table 1. Participants were predominantly male (88.6%), were 43.7 ± 17.0 (SD) years of age (ranged from 18.0 to 76.0) and had a mean BMI of 26.7 ± 5.9 (6.8–42.0). Most SCI participants had motor complete injuries (77.8%). SCI participants were more likely to be male, were younger, had lower BMI, and had higher PAMM levels than the uninjured controls.

**Table 1:** Participant characteristics

Variable	No SCI (n = 51)	Acute SCI (n = 54)	Total cohort (n = 105)	p
Male, n (%)	41 (80.0)	52 (96.3)	93 (88.6)	0.01
Age (years) [Mean ± SD]	49.6 ± 15.5	38.1 ± 16.6	43.7 ± 17.0	0.0004
BMI (kg/m^2^) [Mean ± SD]	28.8 ± 5.1	24.8 ± 6.0	26.7 ± 5.9	0.0004
PAMM (ng/ml) [Mean ± SD]	1.25 ± 1.17	2.53 ± 1.70	1.91 ± 1.59	< 0.0001
Motor complete, n (%)	N/A	42 (77.8)	42 (40.0)	N/A

### Clinical and demographic factors associated with serum PAMM levels

When considering only the No SCI group in univariate analyses, we found no significant associations with PAMM levels (p = 0.13–0.74, Table 2). When considering only the SCI group, PAMM levels were positively associated with age, were greater in men than in women, and were greater in motor complete compared to motor incomplete SCI. Results were similar when considering the total cohort and PAMM levels were positively associated with BMI. When considering the total cohort in multivariable models adjusting for these factors (Table 3), PAMM levels were no longer significantly associated with gender (p = 0.23) or BMI (p = 0.45). PAMM levels increased 0.99 ng/ml for every year increase in age (p = 0.04). Participants with motor complete SCI had significantly greater PAMM levels than those with No SCI (1.70 ng/ml versus 0.94 ng/ml, p = 0.002). Results were similar when considering only the SCI group (p = 0.02 for age and p = 0.04 for gender, data not shown). We found no significant associations with PAMM levels when considering only the No SCI group (p = 0.23–0.67, data not shown) in multivariable models.

**Table 2:** Univariate factors associated with natural log PAMM

Variable	No SCI (n = 51)			Acute SCI (n = 54)			Total cohort (n = 105)		
	β ± SE	e^β^	p	β ± SE	e^β^	p	β ± SE	e^β^	p
Age (years)	− 0.008 ± 0.006	0.99	0.23	− 0.02 ± 0.007	0.98	0.02	− 0.02 ± 0.005	0.98	0.0002
BMI (kg/m^2^)	− 0.03 ± 0.02	0.97	0.13	− 0.009 ± 0.02	0.99	0.64	− 0.04 ± 0.01	0.96	0.01

	ln PAMM (mean ± SD)	e^ln PAMM (ng/ml)^	p	ln PAMM (mean ± SD)	e^ln PAMM^^(ng/ml)^	p	ln PAMM (mean ± SD)	e^ln PAMM^^(ng/ml)^	p
Gender									
Male	− 0.04 ± 0.75	0.96	0.74	0.70 ± 0.80	2.01	0.03	0.37 ± 0.86	1.45	0.03
Female	− 0.12 ± 0.52	0.89		− 0.56 ± 1.03	0.57		− 0.20 ± 0.59	0.82	
SCI severity									
Motor complete	N/A		N/A	0.76 ± 0.78	2.14	0.06	0.76 ± 0.78	2.13*,^^^	< 0.0001
Motor incomplete				0.25 ± 0.93	1.28		0.25 ± 0.93	1.28^^^^	
No SCI							− 0.06 ± 0.71	0.94**	

* p < 0.0001 compared to reference group, ** reference group, ^^^p = 0.04 compared to reference group, ^^^^reference group

**Table 3:** Multivariable factors associated with natural log PAMM

Model p < 0.0001, r^2^ = 0.26, n = 105 (total cohort)			
Variable	β ± SE	e^β^	p
Age (years)	− 0.01 ± 0.005	0.99	0.03
BMI (kg/m^2^)	− 0.01 ± 0.01	0.99	0.40

	Mean PAMM ± SE	e^ln PAMM (ng/ml)^ ± SE	
Gender			
Males	0.36 ± 0.09	1.43 ± 1.09	0.23
Females	0.06 ± 0.23	1.06 ± 1.26	
SCI severity			0.01
Motor complete	0.50 ± 0.18	1.65 ± 1.20*	
Motor incomplete	0.19 ± 0.23	1.21 ± 1.26	
No SCI	− 0.06 ± 0.13	0.94 ± 1.14**	

* p = 0.003 compared to reference group, ** reference group

## Discussion

Here we assessed factors associated with circulating PAMM levels in 54 adults with acute, traumatic SCI and in 51 adult men and women without SCI. Factors associated with PAMM levels varied based on the presence or absence of acute SCI. When adjusting for BMI, age and gender, mean PAMM levels were significantly greater in motor complete SCI compared to uninjured controls (Model p < 0.0001). This model explained 26% of the variation in circulating PAMM levels.

The lack of validated biomarkers of injury severity, neurological recovery, or response to therapy after spinal cord injury is considered a major limitation to both clinical care and to the development of adequately designed and powered clinical trials. The variability in spontaneous neurologic recovery at 1-year post-injury is large and neurological examination does not differentiate between the estimated 30% who experience recovery from those who do not. This is considered a serious limitation to both clinical care and to the development of adequately designed and powered clinical trials. Identification of a novel prognostic biomarker of spontaneous recovery would make clinical trial recruitment more economical and efficient as well as accelerate neuro-recovery research. These results suggest that PAMM may be a biomarker of injury severity in acute SCI. PAMM may also be a novel prognostic biomarker of neurological recovery in acute SCI. That is, PAMM levels tested within the first 3 months after injury might be used to identify those who will experience neurological recovery from those who will not. This hypothesis should be tested in future studies.

We previously reported that PAMM is highly expressed in both white and brown adipose tissue and its expression increases in obesity [8]. Adipocytes are well-known protein secretors, particularly of hormone-like adipokines, which affect several physiological processes, including energy balance and insulin sensitivity [9, 10]. The endoplasmic reticulum (ER) membrane-located PAMM plays a key role in adipocyte biology, notably assembly and secretion of disulfide-bond containing proteins, including adiponectin and collagen isoforms. PAMM is secreted from mature human adipocytes (but not preadipocytes), has anti-inflammatory properties, and suppresses macrophage activation by inhibiting MAPK signaling pathway. PAMM exerts its biological activity by inhibiting signal transduction pathways. However, the PAMM response to various stimuli varies, possibly due to the involvement of several different signaling pathways [11]. Regardless of the signal transduction pathways involved, the mechanism of PAMM action is dependent, at least in part, upon its anti-redox activity. For example, PAMM negatively regulates cytokine-induced inflammatory responses in vascular endothelial cells by suppressing MAPK and NF-kB signal pathways. This suppression was shown to be partially dependent upon the anti-redox activity of PAMM [11]. Not only does PAMM inhibit responses of macrophages and endothelial cells to inflammatory stimuli, but its expression in human atheromatous tissue was nearly 3-fold as compared to normal, reflecting its potential as a therapeutic anti-inflammatory agent for the treatment of vascular inflammatory diseases [11]. The anti-redox activity of PAMM likely also inhibits TNFα-induced MAPK and NF-kB activation through a reduction in ER stress by promoting protein folding and secretion [11]. We recently reported that mutations in the CXXC motif of PAMM suppress its anti-redox activity without suppressing the production of inflammatory cytokines in lipopolysaccharide-stimulated macrophages, indicating that PAMM’s anti-inflammatory and anti-oxidant properties are independent [8].

Mediators of cellular and subcellular oxidative stress have received considerable attention as potential novel neuroprotective targets in acute SCI, including the involved genes, proteins and factors [2, 4, 8, 12]. PAMM is a promising candidate biomarker for SCI because of its dual independent anti-inflammatory and anti-oxidant properties, and because SCI is characterized by both oxidative stress and inflammatory reaction [13–15]. Several other putative inflammatory biomarkers of SCI include interleukins, TGF-B1, insulin-like growth factor 1 (IGF-1) and soluble CD95 ligand (sCD95L). But, there are conflicting reports in the literature about the utility of each as a predictor of inflammatory-related pathologies in SCI (see for a review, [5]). To ensure accuracy and repeatability, these markers have been suggested to be used in parallel with other diagnostic biomarkers for SCI [5]. Additional work is needed to validate these candidate biomarkers prior to implementing use in clinical care.

In this study we report that PAMM is a candidate circulating biomarker of acute, traumatic SCI. We hypothesize that adipose tissue is the source of circulating PAMM. It is unclear why associations between age and gender are stronger in the SCI group compared to uninjured controls, or the mechanisms leading to increased circulating PAMM levels after traumatic SCI. It is possible that increased PAMM expression is an innate native anti-inflammatory response to acute neurotrauma. Also, it is possible that a larger study is required to assess associations between PAMM and clinical or demographic factors, such as age, BMI, or gender, in the general population. Further work is needed to test this hypothesis. Strengths of the current study include the use of a reliable PAMM assay and identification of potential confounding factors, including age. Study limitations include a relatively small sample size with few women. The association between gender and PAMM in acute SCI should be confirmed in a larger study with more women. Despite these limitations, we conclude that PAMM has potential as a novel biomarker of acute SCI. Future research is needed to confirm and validate these findings.

## Conclusions

Our results suggest that PAMM may be a novel biomarker of neurological injury or of native anti-inflammatory responses to neurological injury. More work is needed to establish the role of PAMM and other adipocyte-derived factors in the acute response to neurotrauma.

## Data Availability

The datasets used and/or analysed during the current study are available from the corresponding author on reasonable request.
